# Trachoma in Yunnan province of southwestern China: findings from trachoma rapid assessment

**DOI:** 10.1186/s12886-018-0759-5

**Published:** 2018-04-17

**Authors:** Min Wu, Zhu Lin Hu, Dan He, Wen Rong Xu, Yan Li

**Affiliations:** 1 Department of Ophthalmology, Yunnan Key Laboratory for prevention and treatment of eye diseases, Yunnan Innovation Team for Cataract and Ocular fundus Disease (2017HC010), Yunnan Eye Institute, Yunnan Eye Hospital, The 2nd People’s Hospital of Yunnan Province, Kunming, China; 2 Department of Ophthalmology, The 4th Affiliated Hospital of Kunming Medical University, Kunming, Yunnan China

**Keywords:** Trachoma, Rate, Trachomatous folliculs, Trachomatous trichiasis, Corneal opacity

## Abstract

**Background:**

To understand the situation of active trachoma among children aged 6 to 8 years old and scarring trachoma among those aged 15 and over in Yunnan Province, South-western China.

**Methods:**

A rapid assessment of trachoma was conducted to determine the presence or absence of trachoma in Yunnan. Through risk assessment, 9 sites in 8 suspected trachoma epidemic counties were selected. Trachoma Rapid Assessment was conducted in these areas afterwards. Within each sites, 50 students from grade one in local primary school and adults aged 15 and above with suspected scarring trachoma were examined by survey teams.

**Results:**

A total of 450 children aged 6–8 years and 160 adults aged 15 and above were screened in 9 sites of 8 counties. Only 1 case of active trachoma was found. Detection rate of active trachoma in children was 0.2%(1/450) in all sites and 2% (1/50)in Pingbian County. Out of 150 adults only 1 case of TT and 1 case of CO were found in all the highest at risk communities. People with scarring trachoma were aged over 60 years.

**Conclusions:**

The active trachoma was rarely seen and trachoma is unlikely to be a significant public health problem in Yunnan Province, South-western China.

## Background

It is well known that trachoma is an infectious chronic infectious ocular surface disorder caused by the bacterium Chlamydia trachomatis, and one of the main blinding diseases, especially in Africa. An estimated 2.2 million persons are visually impaired due to trachoma, among which 1.2 million are blind [1, 2]. Conjuctival inflammation is the main characteristic in primary infection of trachoma. As a result of repeated infection, trichiasis and corneal scar may present at late stage which can cause irreversible visual loss [3]. Trachoma is more common in communities with underdeveloped economy, drought and poor sanitation [4, 5]. Data from the Ministry of Health in China showed that the prevalence of trachoma ranged from 63.0%~ 98.0% in 1958 [6]. In the first national survey of blindness and low vision in China, trachoma was the second cause of visual impairment [7, 8]. Trachoma interventions were implemented in China for decades. Following the WHO recommendations to reach the target of WHA51.11 Global elimination of blinding trachoma [9], a trachoma rapid assessment (TRA) was carried out in 14 provinces in P.R.China as a key activity to determine the current situation of trachoma and provide information for future planning. Yunnan province locates in the southwest border of China and is at the far eastern edge of the Himalayan uplift. Yunnan has an area of 394,100 km^2^, 4.1% of the nation’s total, and shares a border of 4060 km with Myanmar, Laos and Vietnam. Mountain land accounts for almost 94% of its total area. The elevation ranges from 76.4 to 6740 m and the average annual rainfall ranges from 584 to 2300 mm. Because of diverse climate and poor traffic conditions, economic development level in Yunnan is relatively backward with more poverty-stricken counties. The prevalence of trachoma was as high as 63.1% in Yunnan Province in 1963 [10]. In 1987, trachoma was reported as one of the three leading causes of blindness in the National survey of blindness in Yunnan. Among 109,181 participants, 107 people were blind caused by trachoma (0.098%) [8]. In 1999, the government of China included Yunnan as one of 12 provinces in which trachoma was still believed to be present [11]. In recent years, trachoma continued to be reported in school health reports [12–14]. Severe drought in recent years led to a shortage of drinking water in 7.42 million people. In the majority of mountainous and the mid-altitude level districts across the province, it is common practice for the whole family to use the same basin of water for face washing. Yunnan Province has been assumed by the government as a region in which trachoma was a public health problem. However, a large scale prevalence survey has so far not been carried out in Yunnan Province and assumptions are commonly based on clinical reports only. As part of a number of TRAs conducted across the country, a TRA was also conducted in Yunnan Province. Purpose of the survey was to assess the presence of active disease, defined as trachomatous inflammation, follicular (TF) and/or trachomatous inflammation, intense (TI) among children aged from 6 to 8 and trachomatous trichiasis (TT) and/or trachoma-related corneal opacity (CO) among those aged 15 and over.

## Methods

### Ethical considerations

The protocol for this trachoma survey was approved by the Ethical Committee of The 2nd People’s Hospital of Yunnan Province, China. During the survey, informed consent was given by every adult and the parents of all enrolled children. The study procedure was explained to the parents, teachers and students by survey staff. The guidelines of the Declaration of Helsinki were strictly followed during the survey.

### Study sites

**Fig. 1:**
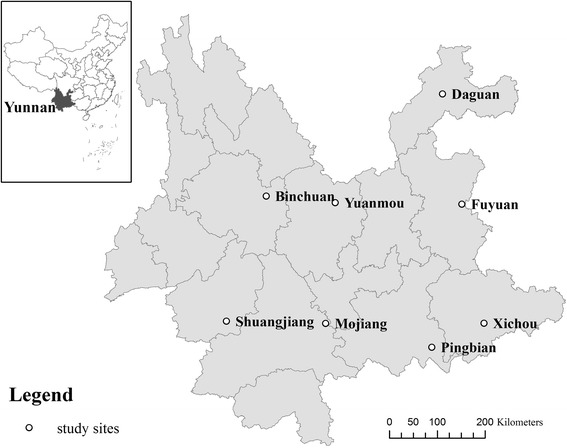
Location of survey area. Up-left figure showed the location of Yunnan Province in China. The eight circles showed the survey areas of TRA in Yunnan Province

The WHO recommended standard methodology for TRA [15, 16] was followed in this survey, including purposive sampling of choosing villages/communities where trachoma is likely to exist. A first phase of investigation was carried out to gather evidence of trachoma, its complications and socioeconomic information in Yunnan. According to the national population census in 2010, the total population in Yunnan Province was 45.9 million. Yunnan Province was divided into 230 districts consisting of populations between 150,000 to 200,000 people. The suspected trachoma epidemic areas were determined within all districts based on the following factors: 1) geographically remote; 2) poor economic development; 3) poor access to water; 4) known high-epidemic and endemic trachoma areas; 5) poor access to health care; and 6) low socio-economic status. Evidence of known endemic trachoma areas, e.g. The number of trichiasis surgical cases performed annually and number of corneal opacity cases seen in recent 5 years, was collected from two sources: literature review and key informants, such as eye doctors, ophthalmic nurses and public health workers. Based on this information, eight counties were identified as suspected trachoma epidemic areas: Daguan, Binchuan, Fuyuan, Mojiang, Shuangjiang, Pingbian, Xichou, and Yuanmou county (See Fig. 1). Nine towns with poor socio-economic status (environmental, sanitation and water supply were also considered) in eight counties were defined as survey clusters.

### Participants

Target population of the survey included the children aged 6 to 8 years for active trachoma and adults aged 15 years and over for blinding trachoma (TT and CO). The gross enrollment rates of primary education in China was estimated as 99.8% in 2014 [17]. Thus for children, a school-based survey was applied. With the help of the local Education Bureau, a list of primary schools in the selected towns was generated and one primary school per town was randomly selected for screening of active trachoma. One class of grade one students in the defined primary school was selected randomly. If the number of students in the class was more than 50, the first 50 students were documented as participants in the survey. When the number of students in the class was less than 50, more students would be recruited from the neighboring class until 50 students were examined. Adult participants were recruited via pre-screening by key informants. One week before the survey team arrived, local village leaders, teachers and village/township doctors etc., were requested to identify adults aged ≥15 years old with trichiasis and corneal opacity door to door. People identified were invited to the township hospital and confirmed by survey team.

### Trachoma grading

Two survey teams were organized and each team included 1 ophthalmologist, 1 field assistant (local ophthalmologist) and 1 driver. One ophthalmologists in each survey team was trained by World Health Organization (WHO) experts during a workshop held by the China Ministry of Health in Beijing. The WHO simplified grading system was taught and applied to identify trachoma. The two trained ophthalmologists led the survey teams. Trachoma was categorized into five grades according to the WHO trachoma grading system [18]: trachomatous inflammation, follicular (TF), trachomatous inflammation, intense (TI), trachomatous trichiasis (TT), trachomatous scarring (TS) and corneal opacity(CO). TF is defined as five or more off-white follicles of 0.5–2.0 mm on the upper tarsal conjunctiva; TI is defined as inflammatory thickening of the upper tarsal conjunctival tissues obscuring more than half the deep tarsal vessels; TS is defined as small scarring in tarsal conjunctiva forming dense fibrotic tissue and distortion of normal lid architecture; TT is defined as at least one eyelash ingrown and touching the globe; CO is defined as corneal opacity which covers part of pupil margin as a result of ingrown eyelashes or secondary bacterial infections. “Active trachoma” was defined as TF and/or TI in either eye. “Scarring trachoma” was defined as TS, TT and/or CO in either eye. A WHO trachoma grading card and slides were carried by survey team.

### Examination of trachoma

After written informed consent was obtained from parents, all the students were examined by trained ophthalmologists using a torch and a 2.5× magnifying binocular loupe. The examiner sat opposite to the student, examining for signs of inflammation (TF and TI) on the upper conjunctiva after turning of upper lid. Between examinations, hand disinfectant was applied to clean the examiners’ hands. Once TF or TI was detected in any student, single dose of Azithromycin was prescribed. Health education information including face washing and strategies against infection was given to students, teachers and parents.

If active trachoma case was detected in non-boarding schools of any survey site, at least 50 children including the siblings of detected case would be examined in the community. If the case was detected in a boarding school, at least 50 students in the same school would be examined for trachoma.

All the adults aged 15 and above screened with suspected TT and/or CO were informed and gathered in the township hospital on the survey day. The survey team examined everyone following the same procedure used in children screening. People with TS, TT and/or CO were documented and people with TT and/or CO were referred to the county hospital for further treatment. Other people with treatable diseases were also referred to the county hospital.

### Data analysis

Double entry of data were performed by two survey staff at the end of each survey day using Microsoft Office Excel 2010(Microsoft Corporation, USA). Integrity of data and consistency check were conducted. Statistical analysis was conducted using SPSS 16.0 software (SPSS Corporation, USA). The percentage of active and scarring trachoma was calculated. The association between risk factors and rate of trachoma was analyzed using Univariate analysis method.

## Results

### Detection rate of trachoma

In total, 450 eligible children and 165 people aged 15 and above were examined in this survey. The response rate in students was 100.0%(450/450) and 96.97% in people aged 15 and above (160/165). The female to male ratio in children and adults examined was 0.95:1 and 1.96:1, respectively. All the children screened were aged from 6 to 8 years old. In adults aged ≥15 years old, the age ranged 15 to 88 years, with a median age of 59 years. In children, 1 case of TF was detected in Pingbian County and oral azithromycin tablets was prescribed. No case of TI was detected. The percentage of TF was 0.2% (95%CI: 0.04–1.2%)(1/450) in total and 2% (95%CI:0.4–10.5%)(1/50) in Pingbian County. In people aged 15 and above, the percentage of TS, TT and CO in “trachoma suspects” was 3.1% (95% CI: 1.3–7.1%)(5/160), 0.6% (95%CI: 0.1–3.5%)(1/160) and 0.6% (95%CI: 0.1–3.5%)(1/160), respectively. The overall detection rate of active trachoma and scarring trachoma was 0.2% and 4.4%, respectively. The detection rate of active and scarring trachoma by survey sites is shown in Table 1. No trachoma was detected in all the remaining survey sites. People detected with TS, TT or CO were aged from 63 to 80 years with median age of 70 years. Within scarring trachoma cases, the CO case was male (14.3%) and other cases of TT or TS were female (85.7%). Although the detection rate of scarring trachoma in females was higher than that in males, there was no statistically significant association between sex and scarring trachoma (*p* = 0.85).

**Table 1:** Detection rate of trachoma in all the survey sites

County	Survey site	No. of children examined	No. of adults ≥15 years old examined	Active trachoma(%)		Scarring trachoma(%)		
				TI(%)	TF(%)	TS(%)	TT(%)	CO(%)
Fuyuan	Dahe	50	29	0(0.0%)	0(0.0%)	3(10.3%)	0(0.0%)	0(0.0%)
Pingbian	Baihe	50	21	0(0.0%)	1(2.0%)	0(0.0%)	0(0.0%)	0(0.0%)
Shuangjiang	Bangbing	50	23	0(0.0%)	0(0.0%)	2(8.7%)	1(4.3%)	1(4.3%)
Other 4 counties	4 sites	300	87	0(0.0%)	0(0.0%)	0(0.0%)	0(0.0%)	0(0.0%)
Total		450	160	0(0.0%)	1(0.2%)	5(3.1%)	1(0.6%)	1(0.6%)

### Further investigation of school where TF case was detected

Since one student with TF was found in Pingbian county, the details of the student were investigated. This student was a 7 year old boy and studied in grade one of a boarding school. The dormitory, water supply and environment were inspected in the school. The school had sufficient clean water supply and children could wash face using clean water every day and take shower once a week. The dormitory accommodated 15 students from different classes of grade one or two. 76 students in grade one and in the same or neighboring dormitory were examined by the survey team. One student with TF was detected in this group and oral azithromycin tablets was prescribed. This student was the first detected TF child’s roommate and shared the same towel. Health education on “F” strategy was given to the children, teachers and parents.

## Discussion

The WHO led Global Alliance for the Elimination of Trachoma (GET2020) aims to elimination the disease as a public health problem in the world by 2020. The key trachoma control strategy is SAFE strategy endorsed by WHO: “surgery” for patients with advanced disease, “antibiotics” azithromycin or tetracycline eye ointment for active trachoma, “facial” cleanliness and “environmental” improvement on water supply and sanitation [4, 5]. In spite of implementation of SAFE strategy, data from Ethiopia [19], Sudan [20], India [21, 22] and countries in Pacific island [23] indicated trachoma is epidemic. The rate of active trachoma ranged from 25.2%–71.0% [19–23]. Trachoma was endemic and the second cause of blindness in China, also in Yunnan Province [6–8, 10]. School health reports showed that the detection rate of active trachoma in primary and middle school students was 29.56% and 18.24% in Fuyuan County in year 2000 [10], and 5.21% in BinChuan County in 2004 [12]. To reduce the trachoma, Ministry of Health in China had launched trachoma intervention programs nationwide since 1960s’. With the intervention of SAFE strategy, findings from several population-based epidemiological studies revealed that trachoma was no longer main cause of blindness in China, including Yunnan Province [24–29]. In our TRA in Yunnan Province, only one case of TF was detected out of 450 6–8 years children and the detection rate of active trachoma was only 0.2% in all the nine survey sites. Hence we conclude that active trachoma is sporadic in Yunnan Province nowadays. The finding is comparable with the findings of very low detection rate of active trachoma from TRA studies in the neighboring Sichuan province, Hainan province and Shandong province [30–32]. The improvement of school environment and general hygiene in community likely contributed to a decrease of trachoma. In the past, almost every village had one primary school. However, most of the schools had very poor infrastructure and insufficient water supply. During the past 10 years, merging of primary schooling had been completed. Small schools in villages were merged into one central school in town. As a result of increased governmental input to the central schools, the school environment, including water supply, was improved.

Out of 450 children and 150 adults only 1 case of TF, 1 case of TT and 1 case of CO were found in the highest at risk communities. Our study proposes that trachoma is unlikely to be a significant public health problem in Yunnan Province. The presence of blinding trachoma in elderly people aged over 60 years could be a sequelae of high epidemic active trachoma in several decades ago. Screening of scarring trachoma suspect people recommended by key informants would be a realistic solution to find people with TT and/or CO who need surgical services. People aged ≥60 years should be the target population for finding scarring trachoma suspect.

TRA is a cost-effective fast survey method recommended by WHO and validated in many countries worldwide. The applications of TRA comprised determining the presence or absence of endemic trachoma, whether or not trachoma is a blinding disease, or prioritizing communities with trachoma for treatment [33]. The TRA was completed in the mountainous region within one month by six survey staffs. It covered 9 sites in 8 counties, which spread in the different directions of Yunnan Province. The longest travel distance was 800 km from the capital city to the survey site. It shows that TRA is an useful tool which can provide an overview of presence or absence of trachoma, even in a mountainous region.

There are some limitations in this study: (1) During screening of scarring trachoma, survey teams did not look for cases using door to door method, but only examined suspected people in township hospitals. There might be selection bias in this process. Some adults with scarring trachoma could be missed when key informants screened in community. For the children, we did school-based survey only. Considering the enrollment rate of primary school in Yunnan Province was 99.5% in 2013, the school children are likely to be good representatives. (2) Microbiological investigation was not conducted in the detected TF case. However the clinical signs in the only TF case fulfilled WHO grading system.

## Conclusion

Active trachoma is a rare condition and scarring trachoma is mainly found in people aged over 60 years old. Trachoma is unlikely to be a significant public health problem in Yunnan Province. Large scale of population-based survey and trachoma prevention program are not necessary. Efforts should be input to find possible blinding trachoma (TT and/or CO) in limited regions and surgical services for trichiasis should be provided when necessary.

## Abbreviations

ASTRA: Acceptance sampling trachoma rapid assessment
CO: Trachoma-related corneal opacity
CRS: cluster random sampling
ENT: Ear, nose & throat
SAFE: Surgery for people at immediate risk of blindness; Antibiotic therapy to treat individual active cases and reduce the community reservoir of infection; Facial cleanliness and improved hygiene to reduce transmission; Environmental improvements to make living conditions better so that the environment no longer facilitates the maintenance and transmission of trachoma
TF: Trachomatous follicular
TI: Trachomatous inflammation intense
TRA: Trachoma rapid assessment
TT: Trachomatous trichiasis
WHO: World Health Organization
